# 3D Genome Engineering: Current Advances and Therapeutic Opportunities in Human Diseases

**DOI:** 10.34133/research.0865

**Published:** 2025-09-01

**Authors:** Xing Jiang, Xiaoli Wang, Song Shen, Shangguo Hou, Chen Yu

**Affiliations:** ^1^ School of Biomedical Sciences and Engineering, South China University of Technology, Guangzhou International Campus, Guangzhou 511442, China.; ^2^ Institute of Cancer Research, Shenzhen Bay Laboratory, Shenzhen 518132, China.; ^3^ Institute of Systems and Physical Biology, Shenzhen Bay Laboratory, Shenzhen 518132, China.; ^4^ Department of Chemistry, College of Sciences, Northeastern University, Shenyang 110819, China.

## Abstract

Dynamic chromatin 3-dimensional (3D) conformation is a key mechanism regulating gene expression and cellular function during development and disease. Elucidating the structure, functional dynamics, and spatiotemporal organization of the 3D genome requires integrating multiple experimental approaches, including chromatin conformation capture techniques, precise genome manipulation tools, and advanced imaging technologies. Notably, CRISPR/Cas systems have emerged as a revolutionary genome-editing platform, offering unprecedented opportunities for manipulating 3D genome organization and investigating disease mechanisms. This review systematically examines recent advances in CRISPR-based mammalian 3D genome engineering and explores the therapeutic potential of 3D genome engineering strategies in disease intervention.

## Introduction

Eukaryotic genomes are highly folded and compressed within micrometer-scale nuclear spaces, forming intricate 3-dimensional (3D) hierarchical structures [1–4]. These structures are crucial for maintaining genome stability, regulating gene expression, determining cellular differentiation trajectories, and phenotypic outcomes [5]. Understanding the principles of organization and function of 3D genomes in space and time is a central theme in cell biology and genomic research [6,7].

**Fig. 1.**
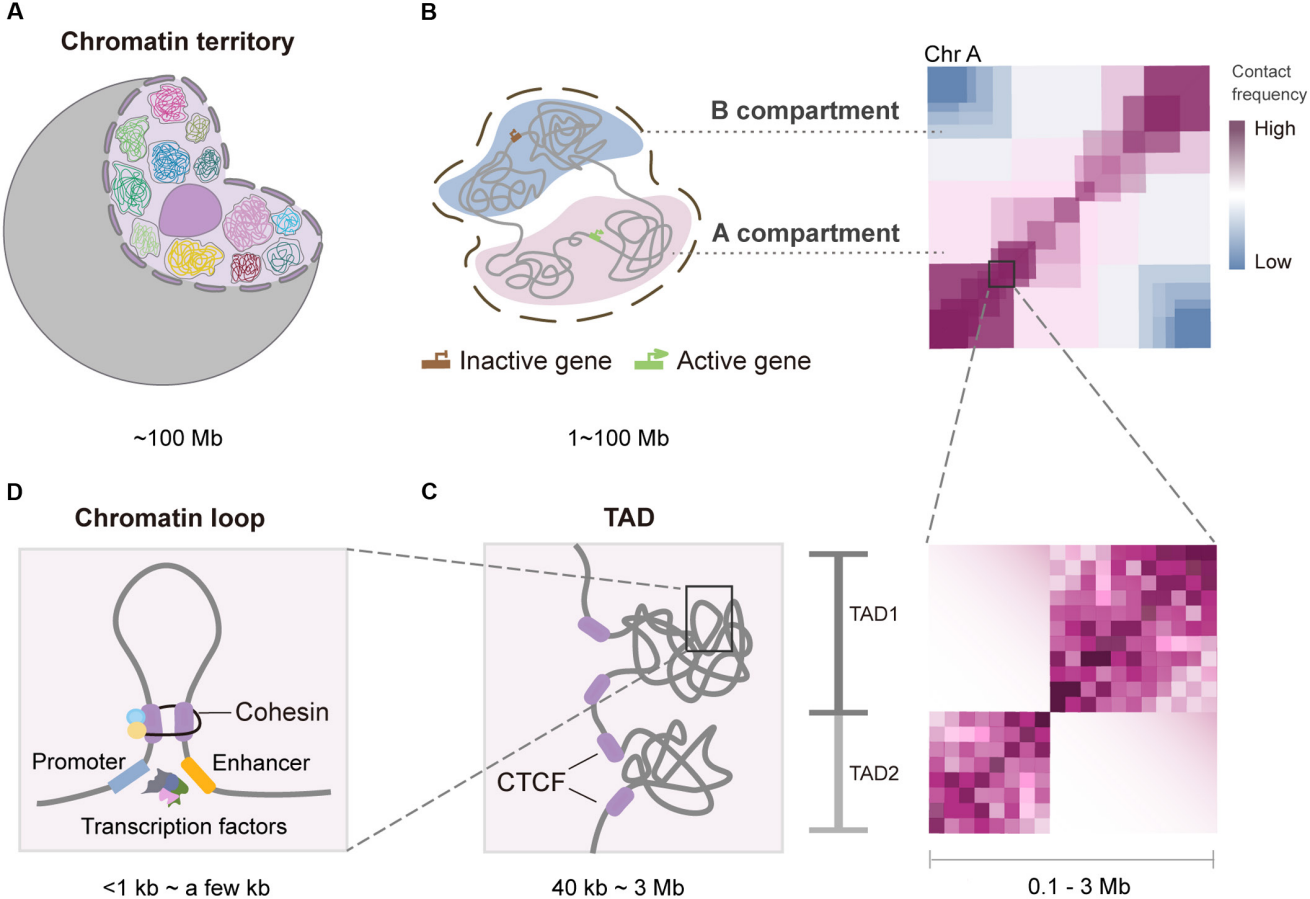
3D genome organization hierarchy. (A) During interphase, individual chromosomes occupy discrete nuclear regions known as chromosome territories. (B) The genome divides into A (open, active, gene-dense) and B (closed, silent, gene-sparse) compartments. In the normalized contact frequency Hi-C heatmap, compartment A (active region) is displayed in pink (high-frequency interactions), while compartment B (silent region) appears in blue (low-frequency interactions). (C) TADs are chromatin subdomains with frequent internal interactions, whose boundaries are typically enriched with insulator proteins such as CTCF, as demarcated by pink rectangles. (D) Chromatin loops are formed through cohesin-mediated extrusion, which stalls upon encountering convergently oriented CBSs. This stabilizes spatial interactions between promoters and distal regulatory elements (e.g., enhancers), facilitating the recruitment of transcriptional machinery and additional regulatory factors.

Early microscopic studies showed that during interphase, each chromosome occupies a specific area of nonoverlapping space known as the chromosome territory (CT) (Fig. 1A) [8–10]. Within CTs, the genome can be divided into spatially segregated A/B compartments [11]. The A compartment is open chromatin, enriched in genes, actively expressed, and rich in histone modifications used for transcriptional activation, usually located in the interior of the nucleus. On the contrary, the B compartment is closed chromatin, inactivated, gene-poor, and compact, contains repressive histone marks, and is located at the periphery of the nucleus (Fig. 1B) [12,13]. This spatial segregation ensures efficient gene regulation and chromatin organization.

Recently, the organizational principles of local genomic regions have begun to be revealed. At a finer scale, regions of chromatin compartments with relatively frequent interactions are defined as topologically associated domains (TADs) (Fig. 1C) [14]. TADs have distinct boundaries between adjacent areas, which are highly enriched in the structural proteins CCCTC-binding factor (CTCF) and cohesin complexes. Chromatin loops form when 2 distant genomic regions physically contact, enabling regulatory elements such as promoters, enhancers, insulators, and silencers to come into proximity to regulate gene expression directly (Fig. 1D) [15,16]. Meanwhile, chromatin loops are more enriched within the same TAD than between TADs, and genes within a TAD are often co-regulated, suggesting the role of TADs as the basic functional unit of 3D genome organization [17].

The mechanisms underlying TADs and chromatin loop formation remain a topic of active investigation, with models such as the loop extrusion hypothesis and liquid–liquid phase separation (LLPS) being proposed [18–20]. While the detailed processes of these models are beyond the scope of this review, engineering strategies have already been successfully applied to 3D genome studies. These approaches have provided valuable insights into the dynamic organization of chromatin in 3D and the interplay between 3D chromatin structures and transcription. These advancements have enhanced our understanding of the causal relationship between the 3D genome and transcription, and revealed new mechanisms underlying genetic diseases and potential therapeutic strategies. In this review, we will highlight the latest progress from an engineering perspective, summarize innovative strategies for 3D genome engineering, and explore their potential applications in disease modeling and therapeutics.

## Genome Engineering to Understand the Principles of 3D Genome Organization

Elucidating the structure, function, and spatiotemporal organization of the 3D genome requires integrating contact mapping techniques, dynamic imaging tools, and chromatin manipulation strategies. Genome engineering tools, particularly CRISPR/Cas systems, enable precise manipulation and analysis of genome sequences, epigenetic modifications, and the formation of specific genomic structures [21]. These tools have opened new avenues for exploring the causal links between 3D genome organization and cellular functions. Here, we highlight recent advancements in programmable 3D genome engineering at multiple scales and discuss how these tools enhance our understanding of the principles and roles of 3D genome organization.

### Chromatin visualization and dynamics tracking

The spatiotemporal organization and chromatin dynamics play a key role in gene regulation. With chromosome conformation capture (3C)-based assays, fluorescence in situ hybridization (FISH), and other technologies, researchers can elucidate the basic principles of chromatin organization on a global scale [22–24]. However, these methods are incompatible with real-time imaging due to sample fixation and DNA denaturation. Visualizing the 4D genome in living cells by incorporating the temporal dimension of 3D chromatin structures is essential for a deeper understanding of cell organization and transcription regulation principles.

**Fig. 2.**
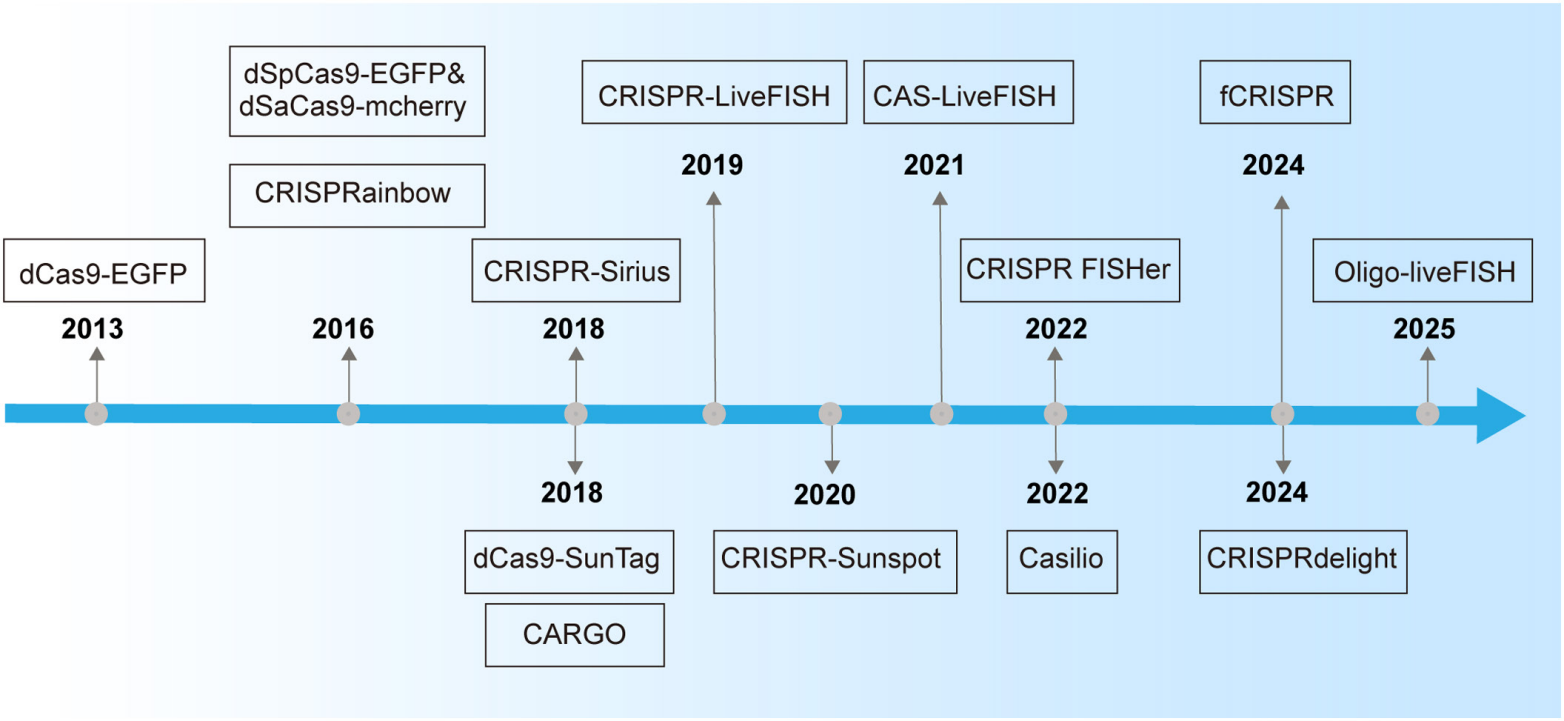
Timeline of CRISPR-based imaging technologies. The field has evolved from early fluorescent labeling systems (e.g., dCas9–EGFP and dual-color fusion proteins dSpCas9-EGFP/dSaCas9-mCherry) to advanced multicolor imaging tools (e.g., CRISPRainbow and CRISPR-Sirius), significantly enhancing multicolor visualization capabilities for genomic loci. Next-generation nonrepetitive sequence imaging technologies, including dCas9-SunTag, CARGO, CRISPR-Sunspot, Casilio, CRISPR-FISHer, and CRISPRdelight, employ strategies such as signal amplification and modular protein recruitment to achieve high-resolution dynamic tracking of single-copy genomic regions. Furthermore, hybrid imaging techniques (e.g., CRISPR-LiveFISH, CAS-LiveFISH, Oligo-liveFISH, and fCRISPR) have expanded the sensitivity and applicability of imaging for investigating 3D genome dynamics in living cells.

**Fig. 3.**
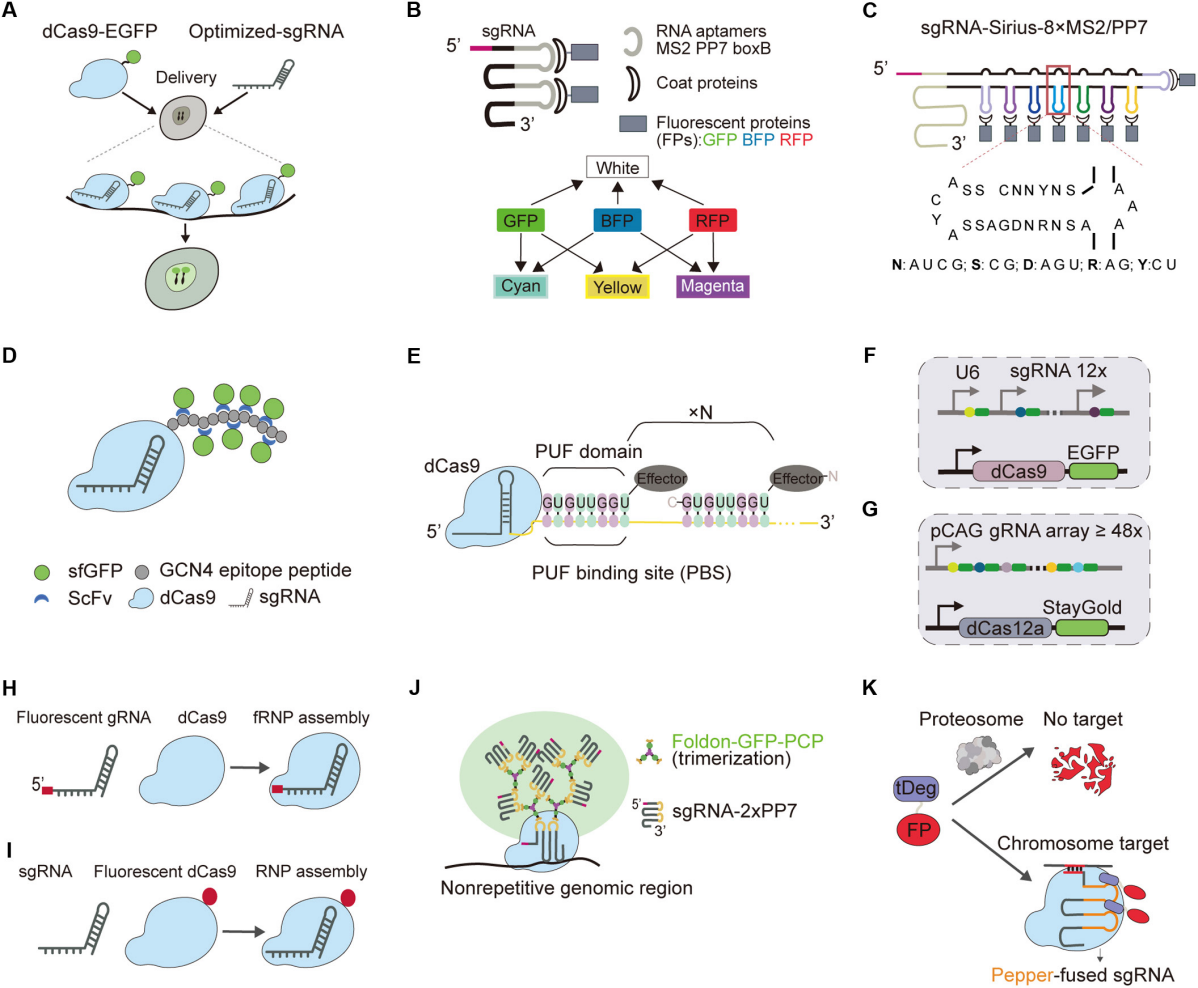
CRISPR-based genomic imaging technologies. (A) dCas9–EGFP. Targets genomic loci via sgRNA guidance, generating sequence-specific fluorescence in live cells. (B) CRISPRainbow. Incorporates MS2/PP7/boxB RNA hairpins into sgRNAs to recruit fluorescent protein (FP) pairs, enabling multicolor imaging via spectral overlap of blue fluorescent protein (BFP)/GFP/red fluorescent protein (RFP). (C) CRISPR-Sirius. Engineered sgRNA scaffold incorporates 8×MS2 or 8×PP7 aptamers in the tetraloop region, enhancing sensitivity for repetitive sequences (≥20 copies) with 2-color labeling. (D) dCas9-SunTag. Amplifying signals via a GCN4 peptide array that recruits multiple scFv–antibody–FP conjugates enables single-copy loci imaging. (E) Casilio. Utilizes sgRNA-PBS sequences to multimerize PUF domain–FP fusions, creating localized signal hubs for nonrepetitive locus imaging. (F) CARGO. Employs a triple-plasmid system (12 sgRNAs per plasmid) for efficient and precise imaging of nonrepetitive genomic regions. (G) CRISPRdelight. CRISPR arrays encode dozens of crRNAs for parallel labeling nonrepetitive sites, achieving high-throughput live-cell imaging. (H) CRISPR-liveFISH. Integrates CRISPR targeting with FISH technology, using fluorescent sgRNA–dCas9 complexes for live-cell genomic locus visualization. (I) CAS-liveFISH. Directly image genomic loci through fluorescently labeled dCas9 proteins, providing an alternative live-cell imaging strategy. (J) CRISPR FISHer. Recruits foldon–GFP–PCP fusion proteins through sgRNA-2×PP7, inducing phase separation to form micrometer-scale condensates at target loci. (K) fCRISPR. TDeg-fused FPs are proteasomally degraded unless stabilized by binding to the pepper aptamer on target-bound sgRNAs, enabling background-free imaging.

Natural chromatin organization can be revealed by directly labeling and imaging endogenous genome sequences in living cells. Several advanced imaging modalities have been engineered to achieve this capability (Fig. 2). Chen et al. [25] reported a combined dCas9–EGFP (enhanced green fluorescent protein) imaging system with optimized single-guide RNA (sgRNA) to visualize repetitive sequences in the telomere and *Mucin* genes, offering the possibility of studying natural chromosome conformation and dynamics in living cells (Fig. 3A). Although this pioneering study provided insights into chromosome dynamics during mitosis, simultaneous imaging of multiple sites and nonrepetitive sequences remained challenging.

The initial breakthrough in multiple genomic loci labeling was achieved through dual-color labeling of DNA loci using dSpCas9-EGFP (*Streptococcus pyogenes*) combined with dSaCas9-mCherry (*Staphylococcus aureus*), which enabled the simultaneous visualization of 2 distinct genomic regions [26]. Building upon this foundation, CRISPRainbow introduced an innovative color-mixing strategy, enabling simultaneous labeling of up to 6 chromosomal loci. This system not only demonstrated the capability to detect repetitive sequences with ≥100 copies but also revealed distinct dynamic properties among different loci in live-cell environments (Fig. 3B) [27]. Subsequently, CRISPR-Sirius markedly improved detection sensitivity through sgRNA scaffold optimization by incorporating 8×MS2 or 8×PP7 aptamers in the tetraloop region. This modification enabled visualization of repetitive sequences with copy numbers as low as 20 while maintaining clear 2-color labeling (Fig. 3C) [28].

For nonrepetitive sequence labeling, the SunTag system employs a polypeptide array design to amplify fluorescent signals, enabling visualization of specific nonrepetitive sequences when coupled with dCas9 (Fig. 3D) [29,30]. The CRISPR-Casilio platform integrates PUF domains fused to effector proteins, which are multimerized at the PUF RNA binding sites (PBS) on the sgRNA scaffold. This design facilitates localized enrichment of effector proteins or fluorescent tags, allowing cost-effective microscopic imaging of nonrepetitive genomic loci marked by a single sgRNA (Fig. 3E). By developing dual-color Casilio probes, this system visualized dynamic interactions between DNA elements in single living cells in the presence or absence of RAD21. Imaging results revealed that upon RAD21 depletion, the distance between the IER5L gene promoter and its super-enhancer increased, while the interaction frequency between 2 RAD21-independent super-enhancers (enriched with H3K27ac) was enhanced. This approach provides novel insights into chromatin structural regulation by capturing dynamic genomic interactions [31].

The CARGO system utilizes a 3-plasmid expression setup (each containing 12 sgRNA expression cassettes) to track enhancer–promoter (E–P) dynamics during live embryonic stem cell (ESC) differentiation quantitatively (Fig. 3F) [32]. In contrast, the dCas12a-based CRISPRdelight system was engineered to generate CRISPR arrays composed of dozens of CRISPR RNAs (crRNAs) for nonrepetitive locus labeling (Fig. 3G). Compared to CARGO, CRISPRdelight offers greater simplicity and lower cost, as it requires shorter CRISPR arrays and fewer crRNA expression plasmids [33].

Among the imaging tools involving “FISH”, CRISPR-LiveFISH achieves high-resolution genomic DNA imaging across diverse human cell types, including progenitor cells, by delivering in vitro-transcribed, fluorescently labeled sgRNA complexed with dCas9 as fluorescent ribonucleoprotein (fRNP) particles (Fig. 3H). This technique demonstrates significant diagnostic potential, enabling the precise detection of chromosomal abnormalities (e.g., Patau syndrome in prenatal amniotic cells) and the tracking of multiple genes in human T lymphocytes. By integrating the Cas9 and Cas13 systems, CRISPR-LiveFISH further accomplishes simultaneous visualization of genomic DNA and RNA transcripts in living cells [34]. Although LiveFISH improves the signal-to-noise ratio (SNR) and enables observation of chromatin dynamics across cell types, its applicability to nonrepetitive sequences remains unverified. In contrast, Oligo-LiveFISH—designed with a nucleic acid pool—overcomes this limitation by flexibly labeling repetitive and nonrepetitive genomic loci, facilitating chromatin dynamics studies in living cells. By integrating Oligo-LiveFISH with 3D super-localization microscopy (SL), researchers can track nonrepetitive genomic regions at 20-nm spatial and 50-ms temporal resolution, capturing real-time enhancer and promoter dynamics [35]. Additionally, CAS-LiveFISH employs in vitro-transcribed sgRNAs and fluorescently tagged dCas9 to monitor specific genomic loci in live mammalian embryos (Fig. 3I). This approach revealed the exceptional in vivo stability of preassembled fRNPs and enabled continuous tracking of locus dynamics during a critical 24-hour window in early mouse embryonic development [36]. Meanwhile, CRISPR FISHer leverages phase separation-mediated local signal amplification to robustly visualize endogenous nonrepetitive sequences with an exceptional SNR, significantly enhancing live-cell imaging resolution (Fig. 3J) [37].

Conventional CRISPR imaging techniques often suffer from high background noise due to constitutive fluorescent protein expression. Zhang et al. [38] developed fCRISPR to overcome this limitation, incorporating a Tat peptide-derived degron domain (tDeg). Here, tDeg-fluorescent proteins are rapidly degraded by cellular proteasomes but stabilize upon binding to Pepper aptamers. By fusing Pepper aptamers to sgRNAs, the system forms a ternary dCas9:sgRNA:fluorescent protein complex, enabling targeted locus-specific labeling (Fig. 3K). This tool effectively minimizes background noise, facilitates real-time chromosome dynamics and length tracking, and monitors critical cellular processes such as DNA double-strand breaks (DSBs) and subsequent repair.

Genomic DNA imaging in living cells allows us to understand DNA’s spatial and temporal organizational principles, offering insights into crucial biomedical processes such as gene expression regulation, DNA replication, and repair. We anticipate further improvements in the labeling resolution and throughput of dynamic DNA tracing tools, which may be achieved by leveraging new signal amplification modules, DNA targeting strategies, and super-resolution microscopy technologies. Such advancements could facilitate genomics, epigenetics, and cell biology research, enabling the dissection of information encoded beyond the DNA sequence in a broader range of biological contexts.

### TAD engineering

CTCF is a multifunctional protein that acts as a transcriptional activator, deterrent, or insulator. It plays a pivotal role in defining TAD boundaries, maintaining chromatin loop structures, and regulating gene expression [39,40]. Its insulating strength at TAD boundaries depends on 2 key features: the orientation of its binding motifs and the number of tandemly arranged CTCF binding sites (CBSs). Studies using CRISPR-mediated knockdown of CTCF have demonstrated that the orientation of the motif critically modulates boundary function. For instance, the outward-facing CTCF site at the right border of the *Protocadherin* (*Pcdh*) TAD prevents aberrant chromatin interactions, maintaining proper chromatin conformation and gene expression within the TAD. Similarly, knockout experiments at the human *HOXD* TAD revealed that 2 outward-facing CTCF sites synergistically reinforce boundary insulation [41].

Beyond orientation, the quantity of CBS clusters determines insulation capacity. Engineered insertion of single or multiple CBS can establish synthetic chromatin domain boundaries and fine-tune E–P interaction frequencies. The study demonstrates that inserting 2 or more CBSs between the *Sox2* gene and its super-enhancer significantly enhances insulation efficacy compared to a single CBS [42]. Recent work combining CRISPR-mediated CTCF depletion with live-cell imaging has uncovered its broader structural roles. CTCF loss disrupts interphase nuclear morphology and induces mitotic defects, including metaphase arrest, anaphase segregation failures, and tripolar spindle formation, highlighting its dual functions in nuclear organization and segregation [43] (Table 1).

**Table 1.** CRISPR-based 3D genome engineering tools. “Reversible” (with asterisks* for conditional reversibility).

Tool name	Core modules	3D genome application scope	Reversibility	Application
CRISPR-KO, e.g., [41]	Cas9 + sgRNA	TAD/loop disruption via CTCF/cohesin depletion	No	Mechanism + Therapeutic potential
CRISPR-KI, e.g., [42]	Cas9 + HDR template	TAD boundary reconstruction by insulator insertion	No	Mechanism + Therapeutic potential
CRISPR-ZIPPER [76]	dCas9–leucine zipper	Artificial chromatin loop formation (forced genomic looping)	No	Mechanism + Therapeutic potential
CLOuD9 [77]	ABA–ABI–PYL1 system	Chemically inducible chromatin looping (ABA-dependent)	Yes	Mechanism + Therapeutic potential
LADL [78]	CIBN–CRY2 optogenetic	Light-controlled chromatin looping (spatiotemporal precision)	Yes	Mechanism + Therapeutic potential
BPCL [79]	SPAAC/SPIEDAC chemistry	Click chemistry-driven forced chromatin looping	Yes	Mechanism + Therapeutic potential
CRISPR-ECHO [105]	ABA–ABI–PYL1 system	Chromatin spatial repositioning (subnuclear compartment targeting)	Yes	Mechanism
CRISPR-GO [106]	ABA–ABI–PYL1 system	Locus-specific nuclear membrane tethering	Yes	Mechanism
CRISPR-PIN [107]	Dockerin–cohesin	Genome locus-specific relocation to the nuclear periphery	No	Mechanism
CRISPRi [135–138]	dCas9–KRAB/DNMT3A	Epigenetic silencing to modulate 3D structure	Yes*	Mechanism + Therapeutic potential
CRISPRoff [139, 140]	DNMT3A–DNMT3L–dCas9–KRAB		Yes	Mechanism + Therapeutic potential
CRISPRa [141]	dCas9–TET1/VPR	Epigenetic activation to reshape 3D architecture	Yes*	Mechanism + Therapeutic potential

**Fig. 4.**
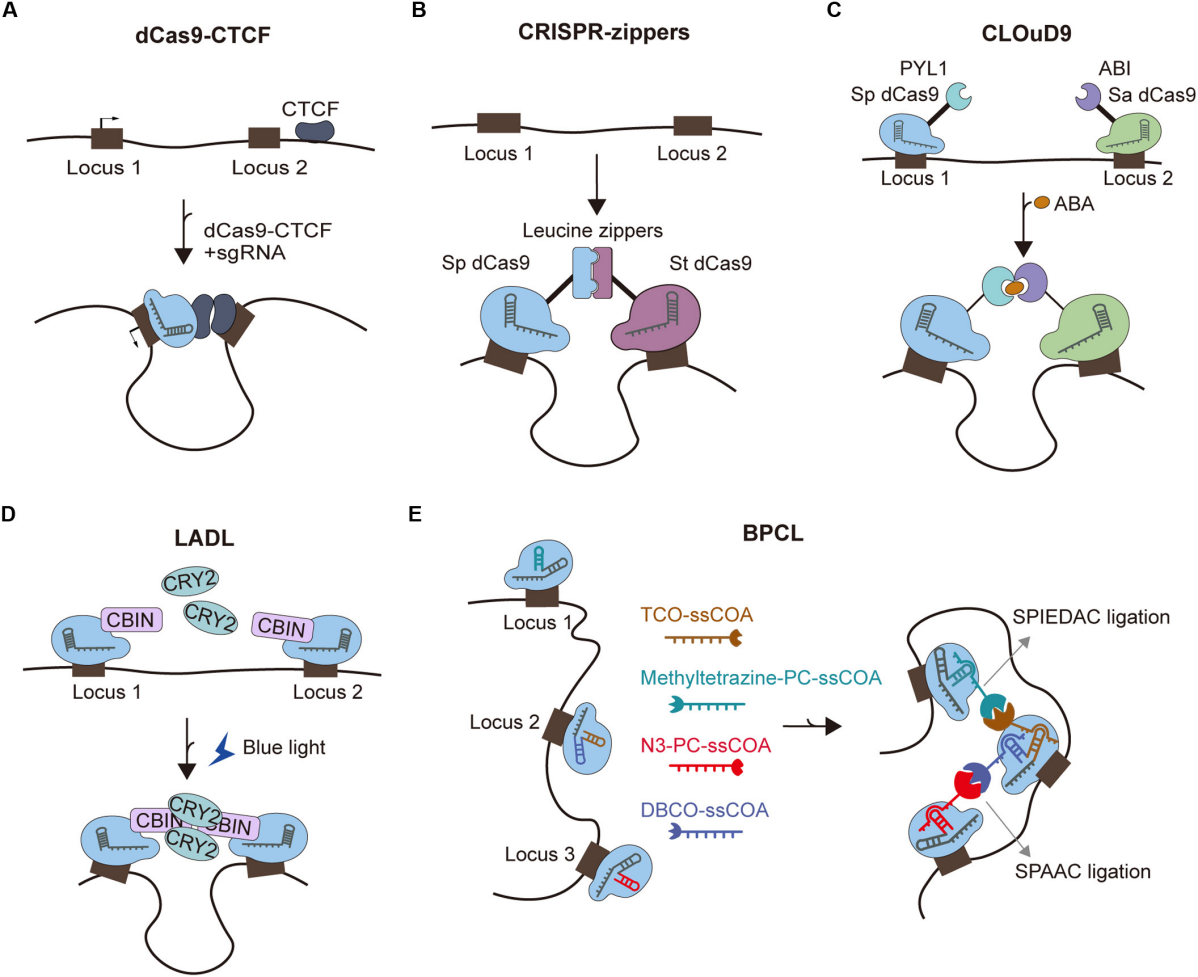
CRISPR-based chromatin loop manipulation tools. (A) dCas9-CTCF. Artificial tethering of CTCF to a gene promoter induces chromatin loop formation and facilitates distal element-dependent transcription. (B) Heterodimer-guided looping. Engineered SpdCas9 and StdCas9 proteins fused to leucine zipper domains create programmable chromatin loops between 2 endogenous genomic loci through targeted protein dimerization, enabling precise spatial control of genomic interactions. (C) CLOuD9. ABA-inducible heterodimerization between SpdCas9-PYL1 and SadCas9-ABI creates locus-specific loops with temporal control. (D) LADL. The 470-nm light triggers dCas9-CIBN/CRY2 heterodimerization, followed by CRY2 oligomerization to stabilize loops between targeted regions. (E) BPCL. Through sgRNA engineering to incorporate specific adapter target sequences, each dCas9/sgRNA complex pair is designed with modified adapters containing matched, discrete bioorthogonal reactive groups. By employing 2 compatible bioorthogonal reaction systems (SPAAC and SPIEDAC), this platform allows for dynamic control of chromatin loop formation at multiple loci.

CTCF’s regulatory impact is evidenced by studies manipulating its binding. Rapid depletion using an auxin-inducible degron (AID) system in mouse ESCs (mESCs) reduced E–P and promoter–promoter (P–P) interactions, dampening gene expression. Conversely, tethering dCas9-CTCF fusions near mutated promoters partially restored target gene expression, confirming that CTCF facilitates long-range enhancer-dependent transcription across diverse cell types (Fig. 4A) [44]. These findings collectively demonstrate that TAD engineering—via targeted CTCF recruitment or deletion—provides a powerful tool to dissect TADs’ role in transcriptional regulation [45].

Meanwhile, the causal relationship between TAD formation and transcription remains highly debated. TADs are proposed to establish a regulatory environment that facilitates enhancer function while preventing ectopic activation of promoters by enhancers from adjacent TADs. For instance, CRISPR/Cas9-mediated deletion of the *TAL1* neighborhood boundary in human embryonic kidney (HEK-293T) cells significantly increased contact frequencies between intra- and extra-neighborhood DNA regions, resulting in 2.3-fold activation of *TAL1* gene expression. This finding demonstrates the critical role of neighborhood integrity in maintaining *TAL1* silencing [46]. Additionally, inversion of CBS elements within the *Pcdh* enhancer reconfigures the topological structure of chromatin loops between distal enhancers and target promoters, consequently altering gene expression patterns [47]. Furthermore, clinical studies have linked CTCF mutations to congenital cardiomyopathy. Specifically, the R567W homozygous mutation (CTCF ^R567W/R567W^) disrupts cardiac-specific TAD architecture, impairing E–P interactions at the Lmod2 locus. This leads to dysregulation of genes critical for normal cardiac development [48].

While these examples illustrate the significant impacts of genomic topological alterations on gene expression, recent studies have supported the TAD–transcription decoupling theory [49–53]. Firstly, a series of studies found temporal dissociation between structural and transcriptional changes. For example, a study using AID systems in mESCs achieved acute CTCF depletion, revealing that while local genomic folding was substantially altered, RNA-sequencing (RNA-seq) analysis detected minimal transcriptomic changes in most genes within 48 h [49,54]. This temporal dissociation was further supported by systematic analyses in *Drosophila melanogaster*, where allele-specific Hi-C and RNA-seq of embryos with highly rearranged balancer chromosomes showed that only several hundred genes exhibited moderate expression changes despite extensive genome reorganization, indicating topology sensitivity in only a subset of genes [55]. Multi-omics data integration further suggests that acute CTCF depletion disrupts global chromatin interactions and accessibility without causing significant transcriptional alterations [56–58]. Secondly, targeted perturbations of TAD at specific loci yielded similar findings. A study demonstrated that deleting multiple inter- and intra-TAD CTCF sites at the *Sox9-Kcnj2* locus in a developmental in vivo model led to the progressive fusion of 2 adjacent TADs, accompanied by only subtle changes in gene regulation [59]. Similarly, comprehensive manipulations of the *SHH* locus—including internal deletions, CTCF site deletions, and boundary sequence alterations (deletions/inversions)—revealed no significant impact on *SHH* expression patterns or levels during development [60]. Thirdly, studies using advanced imaging tools—such as quantitative super-resolution stimulated emission depletion (STED) microscopy—have examined nanoscale spatial coupling between cohesin and CTCF in living cells, though not involving RNA polymerase II (Pol II) [61]. The observed formation of Pol II superclusters independent of CTCF–cohesin complexes suggests that transcription factories may operate autonomously from the higher-order chromatin architecture they mediate. These findings collectively indicate that while TADs play fundamental roles in chromatin organization, at least at specific temporal and spatial contexts, transcription-regulating chromatin interactions occur independently of TAD structures.

The observed decoupling between TAD structural changes and gene expression arises from biological complexity and technical constraints. At the biological level, the temporal dimension is critical: Ectopic regulatory contacts formed after insulation disruption may require extended periods to manifest measurable transcriptional changes [49]. Secondly, the formation of local loops determining transcription activities may be TAD independent. For example, the effects of CTCF depletion are often mitigated by alternative regulatory circuits, such as cohesin-mediated loops or redundant CBSs [50,58,62,63]. What is more, other compensatory mechanisms such as intrinsic E–P interactions [64], epigenetic buffering through DNA methylation and histone modifications [65], and redundant transcription factor (TF) networks maintain transcriptional stability despite 3D genome reorganization [66].

Technical limitations also compound these biological complexities. Population-level sequencing obscures single-cell variability in both chromatin architecture and gene expression, while RNA-seq’s sensitivity thresholds may miss subtle but biologically relevant transcriptional modulation [67–70]. These factors collectively form a multidimensional explanatory framework for TAD–transcription decoupling, highlighting the need for more refined spatiotemporal analyses integrating multi-omics approaches to fully elucidate the complex relationship between 3D genome architecture and transcriptional regulation.

### Engineering chromatin loops

Chromatin loops are fundamental structural and functional units directly regulating gene expression, primarily through promoter–enhancer interactions. By mediating spatial proximity between distal regulatory elements and target genes, these loops enable enhancers to activate transcription over long genomic distances [71].

Interestingly, enhancers drive transcription and play fundamental roles in shaping 3D genome architecture [72,73]. Transcribed noncoding RNAs from active enhancers (eRNAs) have been found to form R-loops to promote specific chromatin remote interactions between enhancers and promoters. These loops then alter chromatin high-level structures in the 3D genome, which regulate gene expression of protocadherin [74]. Additionally, growing evidence suggests that enhancers maintain extensive chromatin organization. For example, enhancers have been found to recruit cohesive proteins to the flank convergent CTCF sites and reporter gene promoters, forming a deposition to facilitate longer-range chromatin contacts, establish contact structural domains, and enable long-range gene activation. In particular, the location of the enhancer on the genome determines which flanking convergent CTCF sites are selected for cohesin protein arrest and chromatin loop formation across structural domains [75].

Recent advances in genome engineering have enabled the precise manipulation of chromatin loops, providing critical insights into their dynamics and functional consequences. Genome editing tools, particularly CRISPR-dCas9 systems, have been adapted to engineer chromatin loops between endogenous loci. Initial chromatin loop manipulation employs CRISPR-dCas9 heterodimers, created by fusing *S. pyogenes* and *Streptococcus thermophilus* dCas9 variants to leucine zipper (ZIP) domains (Fig. 4B). Alternatively, fusing 2 dCas9 monomers as a single polypeptide also constructs chromatin loops. DNA loop formation efficiency is ~40% and 17% with these approaches at 1.4 and 4.7 kb, respectively. At longer distances, a significant increase in E–P interactions can be achieved by ring multiplexing, using 4 sgRNAs to form 2 dCas9-mediated loops, resulting in ~3-fold activation of reporter gene expression [76].

The construction of reversible chromatin loops has advanced our understanding of chromatin loop function and the dynamics of transcriptional activation. The CLOuD9 tool consists of inactivated SpCas9 and SaCas9 fused to a chemically inducible dimerization system based on the abscisic acid (ABA) receptor. The addition of the ABA ligand induces reversible chromatin loops between any 2 genomic regions targeted by sgRNA (Fig. 4C). This approach successfully increased the frequency of contact between the β-globin locus and the locus control region, leading to an approximately 2-fold difference in β-globin protein gene expression [77]. The light-activated dynamic loop (LADL) system is designed to induce the spatial colocalization of CYR2 and dCas9-CIBN fusion proteins through blue light-induced heterodimerization, resulting in the targeted rearrangement of the 3D genome (Fig. 4D). One advantage of this tool is that it allows the formation of long-distance cyclic interactions on demand as early as 4 h after the application of the inducing stimulus, which allows precise analysis of transcription dynamics mediated by the formation of E–P loops [78].

In addition to manipulating individual chromatin loops, powerful tools that dissect the dynamics and complexity of chromatin folding have also been developed. For example, a Bioorthogonal Reaction-Mediated Programmable Chromatin Loop (BPCL) system was established by combining CRISPR with the bio-orthogonal chemical reaction strain-promoted azide-alkyne cycloaddition (SPAAC) and strain-promoted inverse electron-demand Diels-Alder cycloaddition (SPIEDAC) (Fig. 4E). This tool enabled each pair of dCas9/sgRNA complexes to specifically match discrete biological orthologous reaction motifs modified by junctional protein target sequences doped into the sgRNAs, thereby dynamically regulating the formation of different chromatin loops in the same cell without crosstalk [79].

However, artificially reconstructing precise chromatin interactions remains challenging: Engineered loops often show reduced contact efficiency and transcriptional output compared to native structures. Several strategies could be used to improve the transcription efficiency mediated by the formation of artificial loops. Firstly, specific components of transcription machinery or phase-separated transcription factories could be incorporated into current tools to mimic endogenous E–P contacts better. Secondly, new genome editing tools such as more efficient and multiplex CRISPR-associated system proteins could be used, especially when manipulating loops with longer distances. Moreover, introducing new bio-orthogonal reactions and complex junction designs could also facilitate understanding the principles of loop formation and transcription activation in the future [80].

These technical refinements are critical for therapeutic applications. Disease modeling through complex chromatin contact design could provide insight into the causal relationship between chromatin misfolding and the development of genetic diseases. Such research could establish the foundation for treating diseases by correcting aberrant chromatin folding or reprogramming genomic structures. For example, dysfunctional E–P contacts could be circumvented by forming new E–P junctions to correct genetic defects and alleviate cellular disorders [81,82].

### Genome perturbation reveals key factors in 3D genome organization and stability

Genome perturbation studies have significantly advanced our understanding of the molecular mechanisms underlying the establishment and maintenance of higher-order chromatin architecture. Recent genome perturbation screens have begun identifying a network of TFs, chromatin modifiers, and structural proteins that cooperatively regulate chromatin looping, TADs, and long-range gene regulation.

In one such study, coordinately regulated regions (CRRs) that potentially share gene regulatory mechanisms and exhibit similar expression patterns were systematically classified, leading to the identification of the CRE30 DNA element. The research revealed that the conserved CRE30 element recruits CTCF to various sub-CRR loci, facilitating chromatin loop formation and maintaining compact architecture [83]. Xiao et al. [84] and Ortabozkoyun et al. [85] employed an unbiased genome-wide CRISPR screen and biochemical validation to characterize MAZ (*MYC*-associated zinc-finger protein) as a boundary factor partitioning the Hox gene cluster into insulated domains. Their team demonstrated that MAZ exhibits insulator-like functions both in vitro and in vivo: Analogous to CTCF, MAZ interacts with cohesin complexes (including RAD21). Strikingly, in CTCF-depleted ESCs, RAD21 relocalizes to MAZ binding sites, suggesting that MAZ may possess compensatory barrier activity. Two studies reveal that depletion of the cohesin regulator PDS5A, either individually or in combination with its paralog PDS5B, significantly extends cohesin’s chromatin occupancy, which drives persistent loop extrusion activity, generates abnormally expanded TAD structures, and ultimately disrupts nuclear compartmentalization [86,87]. Posttranslational modifications also play key roles in regulating chromatin loop extrusion and TAD formation. As demonstrated by He et al. [88], the deubiquitinase USP13 serves dual regulatory functions in human cells: It maintains cohesin complex homeostasis and ensures the precise temporal dissociation of cohesin from chromatin during mitotic progression.

TFs shape 3D genome architecture coordinately with CTCF and the cohesin complex. TCF-1 and LEF-1, members of the high mobility group (HMG) Tcf/Lef subfamily, orchestrate genomic architecture across multiple scales, including A/B compartmentalization, TADs, chromatin hubs, and focal chromatin loops. In mature CD8^+^ T cells, these TFs dynamically supervise and modulate the 3D genome structure to ensure precise spatial conformation-dependent gene expression, thereby maintaining normal cellular physiology [89]. In T cell progenitor cells, TCF-1 and CTCF co-occupy specific regions of chromatin, which can change the boundary insulation of TAD and promote the interaction between regulatory elements and target genes, which are initially isolated from each other. Furthermore, TCF-1 mediates the cohesin loading factor NIPBL recruitment into active enhancer regions, promoting cohesin complex deposition onto chromatin and subsequent reconfiguration of long-range chromatin interactions [90].

Early studies to explore gene functions on the 3D genome mainly relied on single-gene perturbation strategies. Recently, novel genome perturbation screening technologies expanded genetic discovery to new dimensions. Among them, a screening platform integrating CRISPR perturbation and single-cell assay for transposase-accessible chromatin with high-throughput sequencing (CRISPR-sciATAC) has enabled the systematic identification of genetic determinants of chromatin accessibility across the whole genome. This technology can not only identify regulatory factors affecting local chromatin openness but also reveal the potential roles of these factors in the organization of the 3D genome [91]. Besides, perturb-tracing technology combines CRISPR screening, a novel cellular barcoding readout method (BARC-FISH), and chromatin tracing methods to construct a high-throughput, high-content, image-based screening platform, which can systematically interrogate regulatory factors of high-order 3D chromatin folding structures. This study has identified dozens of new regulatory factors for chromatin folding at different length scales and revealed new functions of known chromatin remodeling factors. For example, in addition to its known function of promoting local chromatin openness, the adenosine triphosphate-dependent helicase CHD7 has been proven to act as a long-range (>3 Mb) chromatin compaction factor, suppressing gene expression at the global level by promoting the condensation of high-order chromatin [92]. These technological breakthroughs have propelled the research on the regulatory mechanisms of the 3D genome into a new stage of systematic and multi-dimensional analysis.

## 3D Genome Modulation as Disease Modeling Tools and Potential Therapies

Emerging insights into 3D genome organization’s roles in differentiation, transcription, and aging have established connections between its structural abnormalities and disease pathogenesis across genomic scales [93–95]. Pathological variations, such as extensive chromosome rearrangement, transposon DNA integration, structural variation (SV), single-nucleotide polymorphisms, and epigenetic factors, affect the 3D conformation of the genome, leading to transcription dysregulation [96–101]. These variations could be corrected in vivo using genome editing approaches. Alternatively, damaged cells could be edited and transplanted back into patients. This section discusses and envisions the potential applications of 3D genome engineering strategies to model and treat diseases.

### A/B compartment shifting

Higher-order chromatin structures are frequently disturbed in cancer and other pathological states. For instance, compared to the standard cell line MCF-10A, the breast cancer cell MCF-7 genome produced about 12% A/B compartment shift [102]. Similar chromatin alterations have been observed in primary acute myeloid leukemia cells, where a significant proportion of genes exhibited A/B compartment switching accompanied by corresponding changes in gene expression [103]. Furthermore, comprehensive studies across multiple prostate cancer cell lines demonstrated specific patterns of chromatin reorganization. Key genes such as androgen receptor (*AR*)*, WNT5A*, *CDK14*, and those adjacent to *TMPRSS2* underwent transitions from compartment B to compartment A, resulting in transcriptional activation. Conversely, 86 genomic loci, including those encoding cadherins, annexins, and inflammatory mediators, showed compartment switching from A to B, potentially leading to transcriptional silencing [104].

**Fig. 5.**
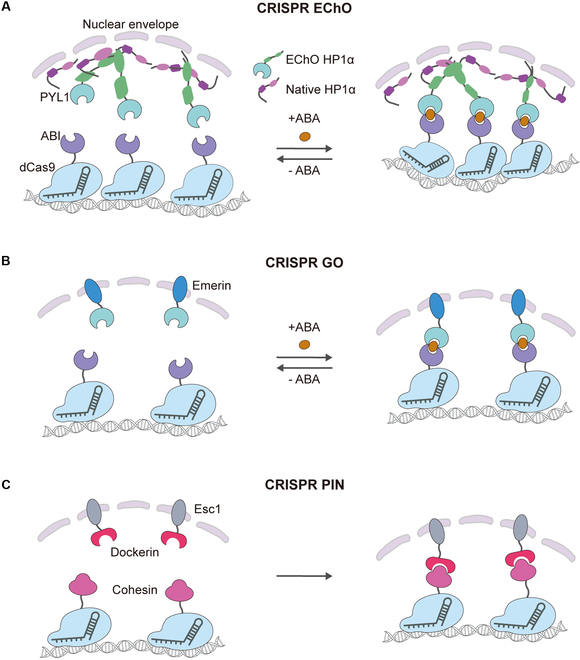
CRISPR-based tools for targeted chromatin tethering. (A) CRISPR-EChO. Without ABA induction, the dCas9-bound tandem repeat region adopts a flexible and open conformation. Introduction of ABA induces dCas9 heterodimerization with engineered HP1α (green). Additional engineered HP1α or endogenous HP1α (magenta) is recruited to the site via HP1α homodimerization and oligomerization. HP1α generates cis-interactions with other sites along the tandem repeat region and trans-interactions with HP1α at different loci within natural heterochromatin or additional dCas9-bound distal sites. These interactions lead to compaction of the target locus, colocalization of multiple targeted loci, and integration with natural heterochromatin. (B) CRISPR-GO. This platform combines an ABA-inducible protein dimerization module with the dCas9 targeting system to achieve reversible subnuclear compartmentalization of specific genomic loci. The system employs ABI–dCas9 fusion proteins for precise gene targeting, while PYL1 is conjugated with the inner nuclear membrane component Emerin. ABA administration induces specific ABI-PYL1 dimerization, leading to directed anchoring of target DNA sequences to the nuclear periphery. This spatial reorganization is fully reversible, with target loci returning to their original nuclear distribution upon ABA withdrawal. (C) CRISPR-PIN. The system harnesses the endogenous Coh-Doc protein interaction network. By engineering dCas9-Coh fusion proteins for locus-specific recognition and utilizing the recruitment capability of nuclear membrane-localized Esc1-Doc, this system enables precise repositioning of target DNA sequences to the nuclear periphery in yeast.

These higher-order chromatin structures could be modified with genome engineering tools. For example, the CRISPR Engineered Chromatin Organization Platform (CRISPR-ECHO) can be used to induce and reversibly bind heterochromatin components in tens of thousands of base endogenous genomic regions (Fig. 5A). This system comprises 3 components: dCas9, an ABA-inducible heterodimerization system derived from ABI-PYL1, and the heterochromatin protein HP1α tagged with superfolder GFP (sfGFP). By targeting tandem repeats, it artificially generates a large and highly concentrated domain of the microscopic heterochromatin protein (HP1α), which forms a new contact with the natural heterochromatin, integrates 2 distant targeted regions, and reversibly changes chromatin from a dispersed to a dense state. This study confirms the multifaceted role of HP1α in shaping the higher-order heterochromatin tissue of eukaryotes, enabling the modeling of chromatin state transitions in disease and a better understanding of pathogenesis at the 3D structural level [105].

Similarly, CRISPR-GO achieves spatial genome reprogramming by fusing dCas9 with subcellular localization proteins. This system also utilizes the ABA-induced ABI-PYL1 system to manipulate the intranuclear positioning of genomic loci dynamically. In particular, when the ABI–dCas9 complex targets specific genomic regions while PYL1 is fused to the nuclear membrane protein Emerin, exogenous ABA addition induces ABI-PYL1 heterodimerization, thereby forcibly anchoring the target locus to the nuclear periphery. This relocalization process becomes reversible upon ABA withdrawal (Fig. 5B) [106]. Furthermore, CRISPR-PIN employs an analogous principle, based on cohesin–dockerin interaction, to recruit dCas9-Coh-bound genomic regions to the perinuclear zone in yeast, thereby expanding the strategic toolkit for 3D genome manipulation (Fig. 5C) [107].

Chaos in 3D genome structure is commonly observed in diseases, particularly in cancer and developmental disorders. Understanding the mechanisms of disease pathogenesis from the perspective of higher-order chromatin organization will provide opportunities to develop novel diagnostic, prognostic, and personalized therapeutic strategies. Simultaneously, such studies necessitate the development of 3D genome editing technologies with enhanced accuracy, precision, and accessibility, which will ultimately pave the way for their future clinical validation.

### Correcting TAD abnormalities

TAD fusion and segregation represent prominent features of cancer genome reorganization. Comparative genomic analyses have revealed significant differences in TAD organization between normal and cancer cells. Specifically, prostate cancer cells exhibit a distinct pattern of TAD alteration, characterized by increased quantity and reduced size compared to their regular counterparts. While normal prostate cells typically maintain TADs averaging approximately 8 Mb, cancer cells demonstrate smaller TADs ranging from 2 to 4 Mb. This structural reorganization is particularly evident at specific genomic loci. For instance, a common deletion at 17p13.1 across the *TP53* tumor suppressor locus results in the bifurcation of a single TAD into 2 distinct smaller TADs [108].

Emerging evidence highlights the critical role of TAD fusion events in disease pathogenesis, particularly through their interaction with super-enhancers to alter gene expression patterns and confer pathological advantages. In T cell acute lymphoblastic leukemia (T-ALL), recurrent TAD fusion events at the *MYC* locus, a key oncogene downstream of NOTCH1 signaling, are consistently observed. These structural alterations, mediated by the loss of CTCF binding, facilitate direct interactions between *MYC* promoters and distal super-enhancers, resulting in significant *MYC* up-regulation and enhanced TAD interactions in T-ALL cells [109]. Additionally, in autosomal dominant adult-onset demyelinating leukodystrophy (ADLD), a rare neurological disorder, a large (∼660 kb) heterozygous deletion upstream of the *LMNB1* promoter disrupts TAD boundaries and boundary elements, resulting in at least 3 forebrain enhancers interacting ectopically with the LMNB1 promoter. This leads to *LMNB1* overexpression and subsequent progressive central nervous system demyelination [110]. Furthermore, in estrogen receptor-positive (ER^+^) breast cancer, 3D chromatin remodeling has been identified as a key mechanism underlying endocrine resistance. Treatment-resistant ER^+^ breast cancer cells exhibit extensive reorganization of chromatin interactions within and between TADs, particularly at active enhancers, promoters, and ER-binding sites. These structural changes lead to altered expression of ER-regulated genes and dynamic remodeling of the ER pathway [111,112]. The findings suggested that changes in 3D chromatin status, altering the spatial structure of regulatory elements and their proximity to cancer-related genes, are one of the fundamental bases of the cancer genome.

The relationship between TAD boundary disruption and gene expression has been extensively studied in many other disease contexts. Studies have been conducted on families with rare limb deformities, showing that different human limb deformities are caused by the deletion, inversion, or duplication of altered *WNT6*/*IHH*/*EPHA4*/*PAX3* loci across TADs [113,114]. It was also demonstrated that chromosomal rearrangements can induce ectopic gene expression and the formation of asymmetric chromatin contact patterns dependent on CTCF anchors and enhancer activity [115]. Additionally, as the core protein of TAD formation, the mutation or loss of CTCF causes severe impacts on organisms [116]. In early mouse embryos, homozygous deletions of CTCF are lethal [117]. Conditional loss of CTCF leads to diverse phenotypic deficits in mice, including impaired spatial learning/memory, fine motor dysfunction, and limb developmental abnormalities, highlighting the causal link between TAD formation disorder and disease [118–120].

**Fig. 6.**
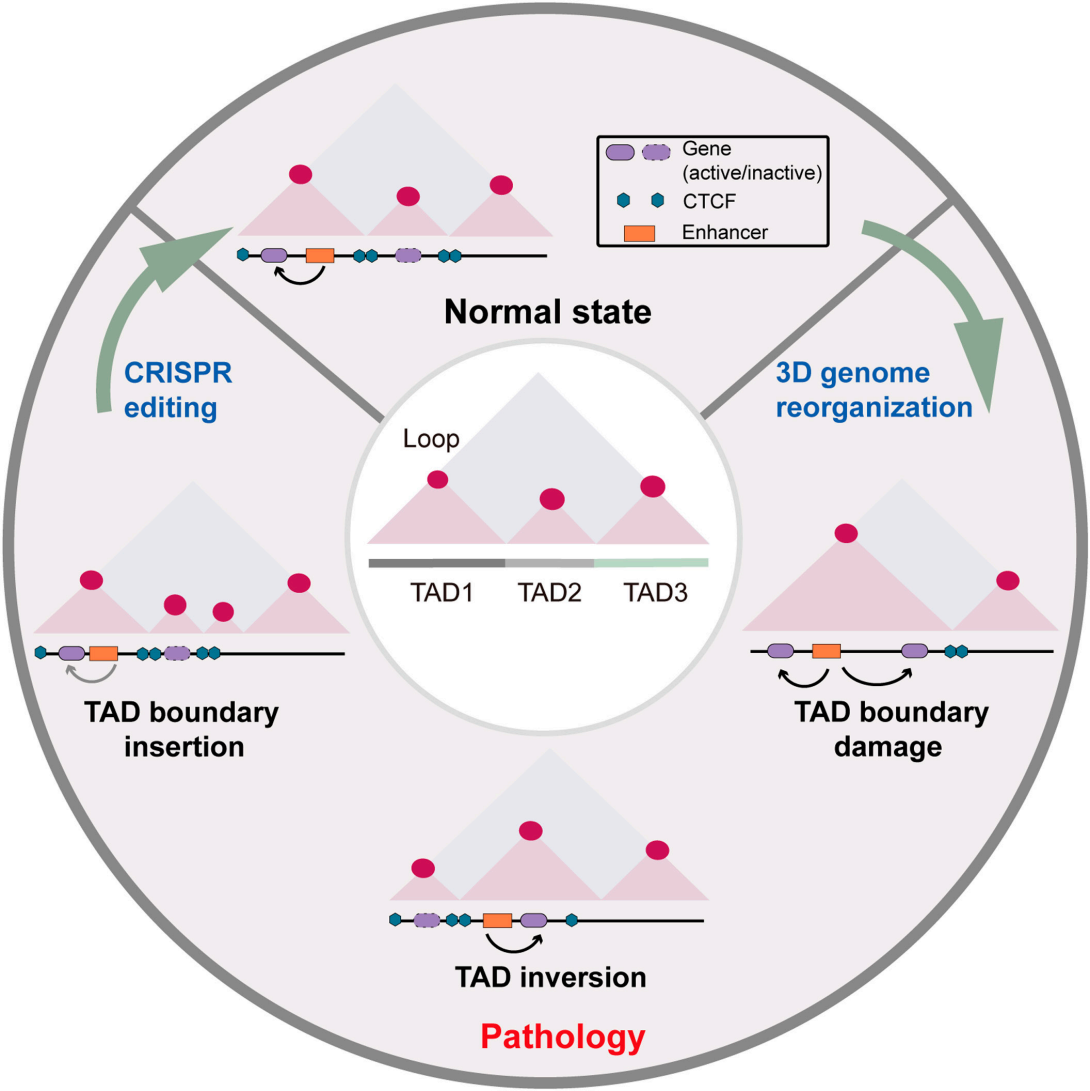
Impacts of TAD boundary aberrations on 3D genome organization and CRISPR-based correction strategies. TAD boundary abnormalities (including CTCF insertions, deletions, and TAD inversions) disrupt the spatial architecture of the 3D genome. The following CRISPR correction strategies can be used: (1) CRISPR-KO for targeted elimination of defective boundaries, (2) dCas9-CTCF fusion proteins to reprogram boundary function by artificial insulator recruitment, and (3) large-fragment insertion techniques to reconstitute chromatin loop structures. These strategies collectively enable the restoration of native TAD topology.

Genome engineering tools could rescue TAD formation disorders (Fig. 6). Unlike conventional genome editing strategies that focus on manipulating individual genomic loci, TAD targeting/correcting provides potential advantages of restoring the coordinated expression of multiple genes within a long range. For example, tandem insertion or deletion of CBS sites could change the insulating strength of domain boundary contact and the size of TADs, thus correcting TAD fusion and segregating events driven by structural variants and expression of genes affected by such events [45]. It has been demonstrated that CTCF is dose dependent in its insulating function at the target locus and TAD. The restoration of CTCF expression restores the correct structure on the altered chromosome [42]. Using dCas9-CTCF to express CTCF at specific sites, TAD boundaries could be re-established, and gene expression could be partially restored [44]. In addition, inversion, deletion, and translocation that cause aberrant TAD formation and gene expression can be modeled or manipulated, which facilitates the analysis of molecular mechanisms of various diseases and the development of gene therapies targeting 3D genome abnormalities.

### Manipulating DNA methylation

DNA methylation is a highly conserved epigenetic modification that plays a crucial role in genome organization and transcriptional silencing. Previous results from single-nucleus methyl-3C sequencing (sn-m3C-seq) have revealed a strong cell-specific correlation between DNA methylation and 3D genome structure, demonstrating their coordinated regulation of gene expression through highly interconnected processes [121,122].

Abnormalities in DNA methylation regulation directly alter gene expression, affect 3D genome structure, and have been widely reported in cancer [123,124]. Human *IDH* mutant gliomas exhibit hypermethylation at the cohesin and CCCTC motifs, resulting in reduced CTCF protein binding, loss of insulation between TADs, and abnormal receptor tyrosine kinase gene PDGFRA activation. Treatment of *IDH*-mutated glioma spheroids with demethylating agents can partially restore insulator function and down-regulate *PDGFRA* [125]. Genome editing tools targeting epigenetic mechanisms, such as CRISPR-DNMT knockout and CRISPR-TET1, could regulate reverse-disrupted chromatin topology in specific disease contexts to offer potential clinical benefits [103,126,127]. Prostaglandin-endoperoxide synthase 2 (*PTGS2*) is frequently overexpressed in various malignancies. Gastric cancer patients with methylated *PTGS2* CpG islands demonstrate significantly improved clinical outcomes, including lower recurrence rates and overall survival [128–130]. Mechanistic studies reveal that methylation-sensitive CTCF/cohesin complexes are enriched near CpG islands associated with *PTGS2* and form chromatin loops at this locus. DNA methylation affects the binding of the CTCF/cohesin complex to the *PTGS2* CpG island and leads to the failure of the enrichment of transcriptional components such as positive elongation factor b at the *PTGS2* motif transcription start site, which in turn leads to the down-regulation of *PTGS2* [131].

Clinically, DNA methyltransferase (DNMT) inhibitors such as decitabine (DAC) and azacytidine (AZA) are approved for the treatment of myelodysplastic syndromes and leukemias [132,133]. However, their therapeutic application is limited by significant toxicity to normal hematopoietic cells [134]. To address these limitations, targeted epigenetic editing tools combining dCas9 with methyltransferases (e.g., DNMT3A and DNMT3L) have been developed for locus-specific methylation modulation and CTCF enrichment regulation [135–138]. Notably, a DNMT3A–DNMT3L–KRAB–dCas9 fusion system has demonstrated long-term reduction of low-density lipoprotein cholesterol levels in transgenic mice and cynomolgus expressing human *PCSK9*, highlighting the therapeutic potential of precision epigenetic editing [139,140]. Fragile X syndrome (FXS) is caused by the silencing of the *FMR1* gene. This silencing is triggered by hypermethylation of a CGG repeat expansion within the gene’s 5′ untranslated region (5′ UTR). Researchers have restored sustained *FMR1* expression in induced pluripotent stem cells (iPSCs) by converting the heterochromatin state to an active conformation using the dCas9-Tet1 demethylation tool [141]. Together, these studies highlight the therapeutic potential of 3D genome structure-correcting strategies using precision epigenetic editing tools.

## Summary and Prospects

The central mission of 3D genome research is to elucidate how the 3D conformation of chromatin orchestrates gene expression, cell differentiation, and disease development. This field provides a novel perspective on the intricate relationship between genome spatial organization and function. Chromatin conformation capture techniques and FISH have been essential for mapping the architecture of chromatin in 3D. The integration of genome engineering empowers researchers to decipher the causal links between genome spatial structure and function. Although CRISPR technology enables precise genome targeting and editing, its application in 3D genome editing remains in its infancy. Further innovation is required to target genome elements more accurately and modify specific chromatin structures efficiently.

A current limitation of 3D genome engineering is its primary focus on modifying pairwise chromatin interactions. Accumulating evidence suggests that multi-way interactions are crucial for chromatin organization and transcriptional regulation. Examples such as the phase separation model of super-enhancer and nested epistasis enhancer networks highlight the importance of multi-way interactions [142,143]. Uncovering these interactions using DNA conformation mapping alone is challenging, given the high complexity and dynamic nature of the 3D genome. On the other hand, engineering approaches offer a direct way to manipulate chromatin structures at multiple sites simultaneously, facilitating a deeper understanding of genomic functionality.

Fully elucidating the causal relationships between 3D genome structure and gene regulation requires not only genome perturbation and transcriptional analysis but also an integrated, multidisciplinary strategy. For instance, when perturbing a specific genomic locus to manipulate local chromatin architecture, how can we distinguish the direct effects of nearby DNA sequences acting as cis-regulatory elements from the indirect consequences of chromatin structural changes? Addressing such challenges demands progress on several aspects. Experimentally, there is a growing need for new synthetic modules that precisely manipulate 3D chromatin structures. For example, tools such as rapid protein degradation systems (e.g., AID) allow temporally controlled perturbations. When coupled with high-resolution live-cell imaging and single-cell multi-omics approaches (e.g., scHi-C and scATAC-seq), these tools enable detailed dissection of the dynamic interplay among sequence variants, chromatin accessibility, and transcriptional outputs at unprecedented resolution. At the computational level, deep learning and artificial intelligence (AI) methods are revolutionizing our ability to analyze and integrate large-scale, multi-modal datasets [144–146]. Such models will enable us to infer regulatory logic, predict chromatin interactions, and quantitatively assess the impact of sequence versus structure on gene expression. Moreover, generative models are increasingly capable of proposing novel perturbations and predicting their likely consequences. These capabilities empower the identification of genomic loci for 3D genome engineering and support mechanistic discovery and translational applications. Rapid progress on both experimental and computational technologies has great potential to fully uncover the causal relationship between 3D genome structure and transcription. This will help us uncover fundamental principles of genome organization and its role in cell identity, plasticity, and disease progression.

In conclusion, 3D genome engineering will continue to drive breakthroughs in the life sciences, opening new frontiers in understanding genome function and regulatory mechanisms. With the integration of multi-omics and AI tools, engineering strategies will lay a robust theoretical and technological foundation for advancing biotechnological innovation and next-generation gene therapies.
